# Evaluation of the genotoxic, antigenotoxic and teratogenic effects of water-based Turkish propolis, and its chemical composition

**DOI:** 10.1039/d6ra03299k

**Published:** 2026-06-04

**Authors:** Semra Araba, Haluk Özparlak, Gökhan Zengin

**Affiliations:** a Selçuk University, Faculty of Science, Department of Biology Selçuklu Konya Turkiye hozparlak@selcuk.edu.tr

## Abstract

Propolis exhibits a wide range of biological properties, including antimicrobial, anti-inflammatory, anti-allergic, antioxidant, anticancer, antitumour and antigenotoxic effects. It mostly contains flavonoids and phenolic compounds. Since propolis is used for therapeutic purposes, its effects should be investigated in detail. This study aims to evaluate the potential genotoxic and antigenotoxic effects of water-based organic Turkish propolis using the Hen's Egg Test for Micronucleus Induction (HET-MN), as well as its chemical composition (by HPLC-DAD). Three different doses (500 µg per egg, 250 µg per egg and 50 µg per egg) of propolis were injected into fertilized chicken eggs at incubation day 8 to determine genotoxic effects. Cyclophosphamide (50 µg per egg) was used as genotoxic agent. In addition, propolis doses were administered together with cyclophosphamide to determine the antigenotoxic effect. Ascorbic acid (50 µg per egg) was used as antigenotoxic agent. Embryonic peripheral blood smears were prepared and stained on 11th day of incubation. The frequencies of micronucleus and nuclear abnormalities in erythrocytes were determined using light microscopy. According to the statistical analysis, water-based organic Turkish propolis did not show any genotoxic effect at the three tested doses. However, only the lowest dose of the propolis showed antigenotoxic effect. In addition, embryos were macroscopically examined for teratogenicity. To evaluate bone development, some embryos were stained with Alizarin Red S, and no teratogenic effects or delays in bone development were observed. Nevertheless, all three propolis doses caused significant decreases in both the number of live embryos and relative embryo weight. HPLC-DAD analysis revealed that benzoic acid was the main component (272.5 µg ml^−1^), followed by caffeic acid (265.4 µg ml^−1^) and ferulic acid (53.8 µg ml^−1^). While the anti-genotoxic effect of low doses of propolis may be attributed to its high antioxidant content, its effect on reducing embryo weight at high doses may be related to its high caffeic acid content. Our findings suggest that low doses of propolis are relatively safe, whereas exposure to high doses could pose potential risks.

## Abstract 2

Our results indicate that lower doses of propolis appear to be generally safe, while higher doses may be associated with possible risks.

## Graphical abstract

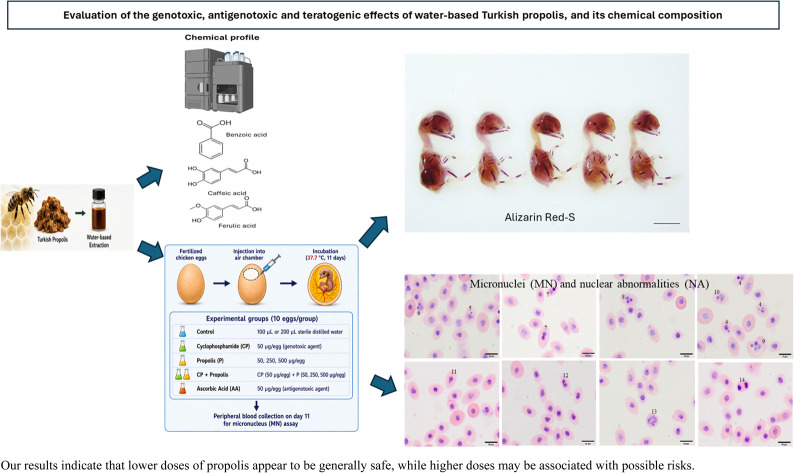

## Introduction

Global epidemiological challenges have increased the significance of natural substances due to their widespread availability and popularity.^1^ In this context, propolis is a resinous mixture collected by honeybees (*Apis mellifera*) from the leaves, buds, and exudates of various plants, partially digested by β-glucosidase present in bee saliva, and subsequently mixed with beeswax.^2^ Used for medicinal purposes since ancient times, propolis is now widely utilized by patients worldwide in various health conditions.^1^ Historically employed as a remedy by the Incas, propolis is currently recognized as an official medicine in the London pharmacopoeia.^3^

The complex chemical composition of propolis is continually evolving due to regional variation. Typically, raw propolis consists of plant resin (45–55%), beeswax (25–35%), essential oils (5–10%), aromatic oils (5%), and pollen, along with other natural components (5%). Moreover, propolis contains various unidentified compounds, including aliphatic acids, esters, aromatic acids, fatty acids, carbohydrates, aldehydes, amino acids, ketones, chalcones, dihydrochalcones, terpenoids, alcohols, vitamins, and minerals. Additionally, its most characteristic components are flavonoids, phenolic acids, and their esters, which play a crucial role in differentiating various types of propolis.^4^ The bioactivity of propolis is often attributed to the synergistic interactions among its components. While antimicrobial properties are the most reported biological activities, propolis has also been documented to exhibit antioxidant, antiradical, antigenotoxic, antitumor, and anti-inflammatory effects.^5^ These multifaceted biological effects underscore the pharmaceutical and medical relevance of propolis, supporting ongoing research and its continued application in healthcare.

Propolis contains more than 500 biologically active components.^6^ Understanding the effects of these components is crucial for human health. In this context, determining the genotoxic and antigenotoxic effects of propolis will contribute to its safe use. The prevalence of genotoxic substances and their associated problems is increasing. To assess the impact of these substances on living organisms, genotoxicity tests have been developed. These tests are also used to evaluate the effects of antigenotoxic substances that repair genotoxic damage. One of the widely used genotoxicity assays is the micronucleus (MN) assay. Wolf & Luepke^7^ reported the formation of micronuclei in peripheral blood erythrocytes of developing embryos from incubated fertilized hen eggs, leading to the development of an alternative method known as the Hen's Egg Test for Micronucleus Induction (HET-MN). This method was subsequently modified and extensively documented in the literature.^8–11^ HET-MN is an extremely simple, cost-effective, and rapid genotoxicity test. It is considered ethically appropriate with respect to animal welfare. The primary advantage of this test is that it is not an animal test, making it more comparable to *in vitro* assays while allowing the tested substance to undergo systemic processes such as absorption, distribution, metabolism, and excretion. In recent years, further studies have refined this method^12,13^ and developed a color atlas of blood cells to aid in evaluation.^14^

Few studies have been conducted to determine the genotoxic and antigenotoxic effects of propolis used therapeutically. In this study, we took a closer look at the potential genotoxic and antigenotoxic effects of a water-based organic Turkish propolis sold under the brand name Fanus®, which is collected from different regions of Turkey. To our knowledge, this is the first time these properties have been evaluated using the HET-MN assay. We used cyclophosphamide (CP) as a genotoxic agent and ascorbic acid (AA) as an antigenotoxic reference. We also explored whether propolis could cause any teratogenic effects in chick embryos. On top of that, we analyzed the chemical makeup of the propolis sample using HPLC-DAD, and then linked those findings to its overall content.

## Material and methods

### Preparation of propolis and test materials

A sample of water-based, organic Turkish propolis (patented Fanus® brand) in 10% liquid form was obtained from the manufacturer (Fanus Gıda ve Organik Ürünler San. Tic. Ltd Şti., Trabzon, Turkiye). The dry matter content of the sample was determined by lyophilization under vacuum at −85 °C for 48 hours. As a result of the lyophilization process, 10 mg ml^−1^ of pure propolis solid material was found in the sample. Based on this data, test doses were prepared by diluting the liquid propolis sample with sterile bidistilled water.

**Fig. 1:**
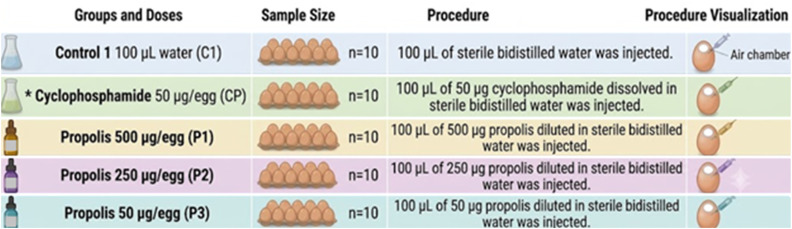
Experimental design I for determining the genotoxic effects of propolis (*A common CP group was established in the experimental designs of Fig. 1 and 2) (generated by Google Gemini).

**Fig. 2:**
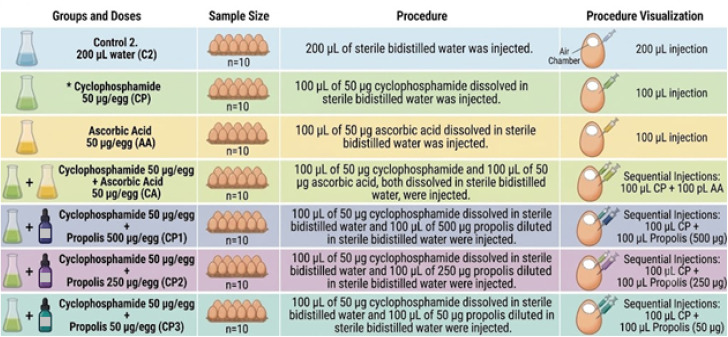
Experimental design II for determining the antigenotoxic effects of propolis (*A common CP group was established in the experimental designs of Fig. 1 and 2) (generated by Google Gemini).

Since there is no existing literature on the effects of propolis on chicken embryos, three propolis doses were selected for this study: 500 µg per egg, 250 µg per egg, and 50 µg per egg (Fig. 1 and 2). CP (C0768; Sigma-Aldrich, St. Louis, MO) (50 µg per egg), dissolved in water, was used as a genotoxic substance, while AA (A92902; Sigma-Aldrich) (50 µg per egg), also dissolved in water, was used as an antigenotoxic substance (Fig. 1 and 2).

### Incubation

A total of 110 fertilized chicken eggs (60.49 ± 2.08 g) from ATABEY hybrid breeder hens were obtained from a commercial enterprise in Konya, Turkiye. The eggs were placed in an incubator (HB500 S; Cimuka, Ankara, Turkiye) maintained at 37.7 °C with 55% relative humidity, where they were automatically rotated 180° once per hour. Approximately 12 hours before injection, the eggs were stabilized with their blunt ends facing upward, and incubation was continued.

### Administration

To determine the potential genotoxic effects of propolis, the experimental design shown in Fig. 1 was established, while the design in Fig. 2 was used to assess its antigenotoxic effects. In both experiments, on the eighth day of incubation, the eggshells were perforated in a sterile cabinet, and the test solutions were injected into the air chambers. Following the injection, the openings were promptly sealed with liquid paraffin, and incubation was continued. To facilitate diffusion of the injected solutions, the eggs were kept upright in the incubator for the first hour.

### Blood sampling and staining

On the 11th day of incubation, the eggs were opened, and the embryos' viability was assessed. For HET-MN, blood samples were collected from the major blood vessels in the peripheral circulatory system of the chorioallantoic membranes of live embryos.^12–14^ Blood smears were prepared immediately after collection, air-dried, and stained using a modified May-Grünwald–Giemsa method.^7,9–11^ The smears were covered with 2 ml of filtered May-Grünwald solution (101424, Merck KGaA, Darmstadt, Germany) for 3 minutes, followed by the addition of an equal volume of 0.1 M disodium citrate/NaOH buffer (pH 5.2) without rinsing the May-Grünwald solution. Upon the appearance of a metallic sheen (but not exceeding 5 minutes), the solution was discarded. After thorough rinsing with demineralized water, a filtered 30% (v/v) Giemsa solution (109204, Merck) in 0.1 M disodium citrate/NaOH buffer (pH 5.2) was applied for 22 minutes. The slides were then thoroughly rinsed with demineralized water, dehydrated in ethanol for 2 minutes, and immersed in xylene (108685, Merck) for 15 minutes before being mounted with coverslips using Entellan® (107961, Merck).^7,9–11^

### Embryo staining

To detect potential skeletal developmental delays, at least three macroscopically normal embryos from each group were examined using Alizarin Red-S staining.^15^ Embryos were fixed in 10% neutral formalin (104002, Merck) for at least one week, then stored in 70% ethanol (100983, Merck) for two days. Subsequently, the embryos were stained for three days in a 0.01% Alizarin Red-S (106278, Merck) solution freshly prepared in 1% KOH (105012, Merck). After staining, the embryos were placed in 2% KOH for five days, followed by immersion in a mixture of 2% KOH, glycerol (Emir Kimya), and ammonia (5432, Merck) (1 : 1 : 1) for one week. Finally, the embryos were stored in glycerol, and macroscopic photographs were taken.

### Scoring of MN

Slides were examined using a light microscope with an immersion objective, and digital images were recorded (DP2-BSW, Olympus Corporation, Ver. 1.2, 2006 digital image analysis program). The rates of nuclear abnormalities (NA) and MN in the peripheral blood erythrocytes of each embryo were determined. NA assessment included abnormalities such as binucleated (binuclei), multinucleated (multinuclei), blebbed nuclei, notched nuclei, and lobed nuclei.^16^ MN assessment was conducted based on the criteria outlined by Wolf & Luepke,^7^ Wolf *et al.*,^10^ and Maul *et al.*^14^ According to these references, MN were identified as round to oval, well-defined, three-dimensional structures with staining properties similar to the nucleus, not exceeding one-third of the nucleus size. The classification of cell types in the embryonic stage was based on the atlas published by Maul *et al.*^14^ According to this atlas, MN were assessed only in polychromatic and normochromatic erythrocytes, as well as NA.

### Phenolic profile

Phenolic profiling was performed using a Shimadzu HPLC system equipped with a diode array detector (SPD-M10A vp DAD, *λ*_max_ = 278 nm), autosampler (SIL-10AD vp), pump (LC-10AD vp), system controller (SCL-10A vp), degasser (DGU-14A), and column oven (CTO-10A vp). Separation was achieved on an Agilent Eclipse XDB-C18 column (250 × 4.6 mm, 5 µm) maintained at 30 °C. The injection volume was 20 µl, and the flow rate was set at 0.8 ml min^−1^. The mobile phase consisted of solvent A (3% acetic acid in water) and solvent B (methanol), following the method described by Caponio *et al.*^17^ A set of reference standards was used for compound identification, including gallic acid, protocatechuic acid, syringic acid, ferulic acid, *p*-hydroxybenzoic acid, catechin, chlorogenic acid, hesperidin, caffeic acid, epicatechin, vanillin, *p*-coumaric acid, benzoic acid, quercetin, *o*-coumaric acid, kaempferol, rosmarinic acid, eriodictyol, rutin, cinnamic acid, and luteolin.

### Statistical analysis

Statistical analyses were performed using the SPSS software package (13.0.0; SPSS Inc., Chicago, IL), with a significance level of *p* < 0.05. The Mann–Whitney *U* test was used to determine differences in MN and NA rates between the control, CP groups, and other experimental groups. The *t*-test was used to assess differences in live and relative embryo weights among the control, CP, and other groups.

## Results

### General observations and Alizarin Red-S staining

**Fig. 3:**
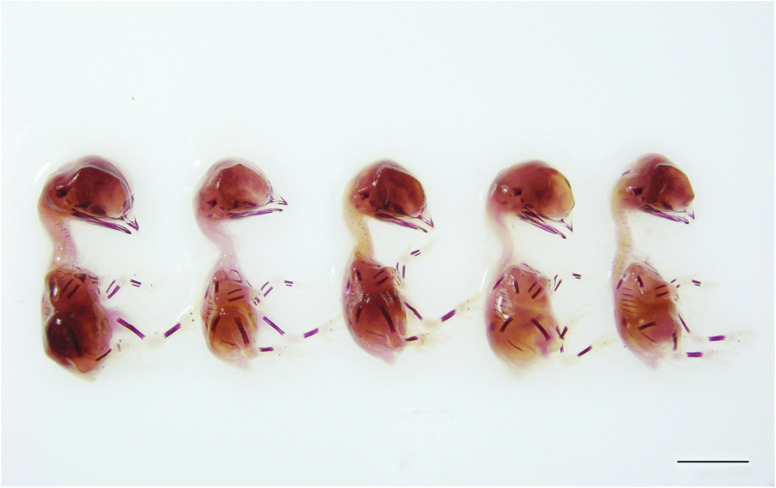
Images of embryos stained with Alizarin Red-S, belonging to the C1, CP, P1, P2, and P3 groups (from left to right) in experimental design I, which was used to determine genotoxic effects (scale bar: 1 cm).

**Fig. 4:**
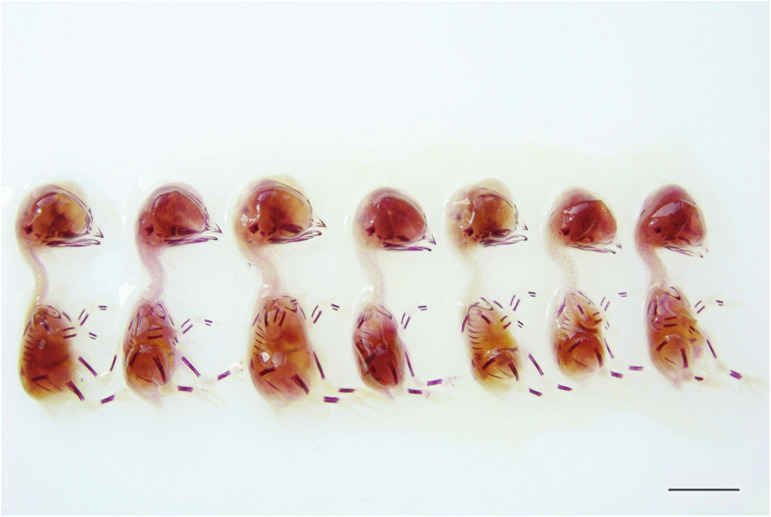
Images of embryos stained with Alizarin Red-S, belonging to the C2, CP, AA, CA, CP1, CP2, and CP3 groups (from left to right) in experimental design II, which was used to determine antigenotoxic effects (scale bar: 1 cm).

The macroscopic appearance of the embryos on incubation day 11 was compatible with the Hamburger–Hamilton scale^18^ and no malformation or growth retardation was observed. When examined macroscopically, the embryos stained with Alizarin Red-S showed no delay or abnormalities in bone development (Fig. 3 and 4).

The live embryo weights and relative embryo weights on day 11 of the CP, P1, P2, and P3 groups were significantly lower compared to the C1 group (*p* < 0.05) (Table 1). Similarly, the live embryo weights and relative embryo weights on day 11 of the CP, CP1, CP2, and CP3 groups were significantly lower compared to the C2 group (*p* < 0.05) (Table 2). The live embryo weight and relative embryo weight on day 11 of the AA group were significantly higher compared to the CP group (*p* < 0.05) (Table 2).

**Table 1:** Data of embryos from experimental design I, which was used to determine genotoxic effects on the 11th day of incubation

Groups	Number of fertilized eggs	Number of viable embryos	Viability rate (%)	Viable embryo weight (g) (mean ± SD)	Relative embryo weight (%) (mean ± SD)	Number of abnormal embryos/abnormal embryo rate (%)
C1	10	10	100	3.34 ± 0.23	5.98 ± 0.42	0/0
CP	9	9	100	2.62 ± 0.18^a^	4.54 ± 0.30^a^	0/0
P1	9	9	100	2.97 ± 0.22^a^	5.26 ± 0.32^a^	0/0
P2	10	10	100	3.00 ± 0.20^a^	5.37 ± 0.34^a^	0/0
P3	7	7	100	2.90 ± 0.32^a^	5.27 ± 0.76^a^	0/0

a The difference compared to the C1 group is statistically significant (*t*-test, *p* < 0.05).

**Table 2:** Data of embryos from experimental design II, which was used to determine antigenotoxic effects on the 11th day of incubation

Groups	Number of fertilized eggs	Number of viable embryos	Viability rate (%)	Viable embryo weight (g) (mean ± SD)	Relative embryo weight (%) (mean ± SD)	Number of abnormal embryos/abnormal embryo rate (%)
C2	10	10	100	2.89 ± 0.18	5.13 ± 0.33	0/0
CP	9	9	100	2.62 ± 0.18^a^	4.54 ± 0.30^a^	0/0
AA	10	10	100	3.00 ± 0.23^b^	5.32 ± 0.51^b^	0/0
CA	10	10	100	2.79 ± 0.21	4.83 ± 0.41	0/0
CP1	8	8	100	2.47 ± 0.14^a^	4.26 ± 0.22^a^	0/0
CP2	7	7	100	2.55 ± 0.10^a^	4.40 ± 0.27^a^	0/0
CP3	8	8	100	2.59 ± 0.15^a^	4.45 ± 0.32^a^	0/0

a The difference compared to the C2 group is statistically significant (*t*-test, *p* < 0.05).

b The difference compared to the CP group is statistically significant (*t*-test, *p* < 0.05).

### HET-MN results

**Fig. 5:**
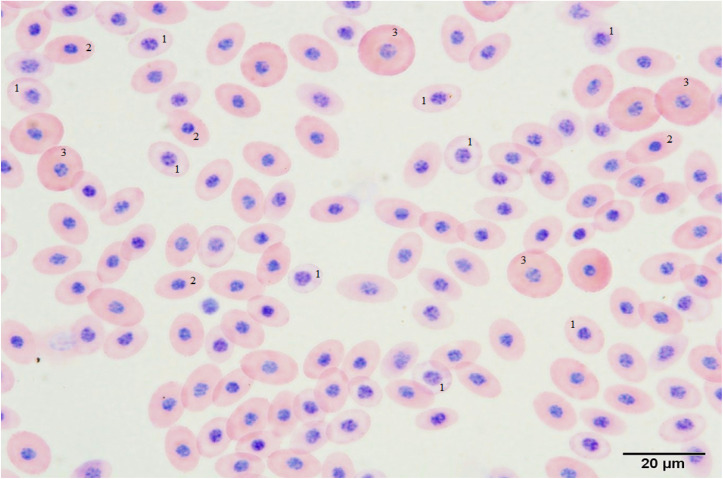
A general observation of a blood smear prepared from an embryo belonging to the control group on the 11th day of incubation: ^1^ polychromatic erythrocyte; ^2^ normochromatic erythrocyte; ^3^ primitive erythrocyte (scale bar: 20 µm).

**Fig. 6:**
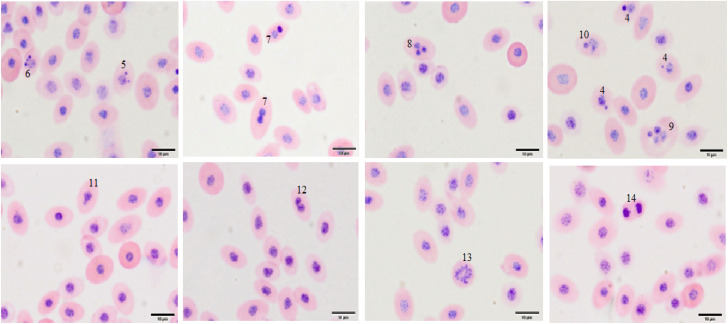
Micronuclei (MN) and nuclear abnormalities (NA) and cell types in blood smears prepared from embryos of different groups on the 11th day of incubation: ^4^ polychromatic erythrocyte with one MN; ^5^ polychromatic erythrocyte with two MN; ^6^ polychromatic erythrocyte with three MN; ^7^ binucleated polychromatic erythrocyte; ^8^ trinucleated polychromatic erythrocyte; ^9^ tetranucleated polychromatic erythrocyte; ^10^ polychromatic erythrocyte with a lobed nucleus; ^11^ polychromatic erythrocyte with a budding nucleus; ^12^ polychromatic erythrocyte with a notched nucleus; ^13^ polychromatic erythrocyte in metaphase; ^14^ polychromatic erythrocyte in anaphase (scale bar: 10 µm).

A high proportion of polychromatic and normochromatic erythrocytes were observed in all blood preparations, while primitive erythrocytes were seen at lower frequencies. Thrombocytes were observed in small amounts. Other cell types, including erythroblasts, early polychromatic erythrocytes, and granular leukocytes, were rarely encountered. Examples of cell types, as well as NA and MN, are shown in Fig. 5 and 6.

The MN, NA, and total abnormality rates for the groups aimed at determining genotoxic effects are presented in Table 3. Compared with the C1 group, the MN, NA, and total abnormality rates in the CP group were significantly higher (*p* < 0.05). In contrast, when the P1, P2, and P3 groups were compared to the C1 group, the differences were not statistically significant (*p* > 0.05) (Table 3). As expected, the CP group exhibited genotoxic effects, whereas propolis did not at any of the three doses.

**Table 3:** HET-MN results for experimental design I, which was used to determine genotoxic effects

Groups	Application volume	Application day/blood sample collection days	Number of blood samples	Number of erythrocytes evaluated per egg	MN rate (%) (mean ± SD)	NA rate (%) (mean ± SD)	Total MN and NA rate (%) (mean ± SD)
C1	100 µl	8/11	9	5000	0.00 ± 0.00	0.01 ± 0.01	0.01 ± 0.01
CP	100 µl	8/11	7	5000	2.29 ± 0.12^a^	0.29 ± 0.06^a^	2.57 ± 0.14^a^
P1	100 µl	8/11	9	5000	0.01 ± 0.01	0.01 ± 0.01	0.02 ± 0.02
P2	100 µl	8/11	9	5000	0.01 ± 0.01	0.01 ± 0.01	0.02 ± 0.02
P3	100 µl	8/11	6	5000	0.01 ± 0.02	0.00 ± 0.00	0.01 ± 0.02

a The difference compared to the C1 group is statistically significant (Mann–Whitney *U*, *p* < 0.05).

The MN, NA, and total abnormality rates for the groups aimed at determining antigenotoxic effects are presented in Table 4. When the CP, CA, CP1, CP2, and CP3 groups were compared with the C2 group, the MN, NA, and total abnormality rates were significantly higher (*p* < 0.05). As expected, the CP group induced genotoxic effects. When the CA and CP3 groups were compared with the CP group, the MN and total abnormality rates were significantly lower (*p* < 0.05). Compared with the CP group, the NA rate was significantly lower in the CP3 group (*p* < 0.05). Antigenotoxic effects were observed in both groups, and AsA and low-dose propolis reduced CP-induced genotoxic effects. When comparing the CP1 group to the CP group, the NA and total abnormality rates increased significantly (*p* < 0.05), indicating that the high dose of propolis enhanced the genotoxic effects of CP, indicating a synergistic effect (Table 4).

**Table 4:** HET-MN results for experimental design II, which was used to determine antigenotoxic effects

Groups	Application volume	Application/blood sample collection days	Number of blood samples	Number of erythrocytes evaluated per egg	MN rate (%) (mean ± SD)	NA rate (%) (mean ± SD)	Total MN and NA rate (%) (mean ± SD)
C2	200 µl	8/11	8	5000	0.00 ± 0.00	0.01 ± 0.02	0.01 ± 0.02
CP	100 µl	8/11	7	5000	2.29 ± 0.12^a^	0.29 ± 0.06^a^	2.57 ± 0.14^a^
AA	100 µl	8/11	7	5000	0.01 ± 0.01	0.01 ± 0.01	0.01 ± 0.02
CA	100 µl + 100 µl	8/11	8	5000	1.52 ± 0.17^a^^b^	0.25 ± 0.08^a^	1.77 ± 0.19^a^^b^
CP1	100 µl + 100 µl	8/11	7	5000	2.45 ± 0.22^a^	0.58 ± 0.29^a^^b^	3.03 ± 0.48^a^^b^
CP2	100 µl + 100 µl	8/11	7	5000	1.95 ± 0.31^a^	0.36 ± 0.07^a^	2.31 ± 0.36^a^
CP3	100 µl + 100 µl	8/11	7	5000	1.85 ± 0.22^a^^b^	0.21 ± 0.04^a^^b^	2.06 ± 0.25^a^^b^

a The difference compared to the C2 group is statistically significant (Mann–Whitney *U*, *p* < 0.05).

b The difference compared to the CP group is statistically significant (Mann–Whitney *U*, *p* < 0.05).

### HPLC results

The HPLC profile findings showed a wide range of phenolic acids and flavonoids, however several components were missing or below the detection limit. According to Table 5, benzoic acid was the predominant compound (272.5 µg ml^−1^), followed by caffeic acid (265.4 µg ml^−1^), ferulic acid (53.8 µg ml^−1^), and *p*-coumaric acid (44 µg ml^−1^). We also found moderate quantities of *p*-hydroxybenzoic acid (8.1 µg ml^−1^), vanillin (8.6 µg ml^−1^), and cinnamic acid (25.6 µg ml^−1^). There were small amounts of gallic acid (4.5 µg ml^−1^), protocatechuic acid (3.6 µg ml^−1^), and flavonoids like luteolin (5.4 µg ml^−1^), kaempferol (4.3 µg ml^−1^), and apigenin (5.5 µg ml^−1^). Eriodictyol was found at a low level (1.1 µg ml^−1^). Conversely, several flavonoids such as catechin, epicatechin, rutin, hesperidin and quercetin were undetected. The chromatographic profile indicated that the extract was mostly enriched with simple phenolic acids, specifically hydroxycinnamic and benzoic acid derivatives, rather than flavonoid glycosides.

**Table 5:** Chemical composition of water-based organic Turkish propolis (µg ml^−1^) (nd: not detected) (mean ± SD of three parallel measurements)

Compounds	Amount	Linear range (mg L^−1^)	Linear equation	*R* ^2^	LOD (mg L^−1^)	LOQ (mg m^−1^)
Gallic acid	4.5 ± 0.05	0.20–25.0	*y* = 64 487*x* − 15 309	0.9993	0.07	0.23
Protocatechic acid	3.6 ± 0.03	0.20–25.0	*y* = 48 107*x* − 11 153	0.9991	0.09	0.26
*p*-Hydroxy benzoic acid	8.1 ± 0.2	0.20–25.0	*y* = 62 896*x* − 11 801	0.9994	0.007	0.02
Chlorogenic acid	1.8 ± 0.1	0.35–45.0	*y* = 37 172*x* − 20 503	0.9988	0.08	0.24
Caffeic acid	265.4 ± 6.3	0.16–21.0	*y* = 10 1382*x* − 18 712	0.9993	0.05	0.16
Vanilin	8.6 ± 0.3	0.08–10.0	*y* = 15 3084*x* − 7178.3	0.9995	0.02	0.06
*p*-Coumaric acid	44 ± 1.7	0.04–6.0	*y* = 17 5872*x* − 5464.3	0.9996	0.07	0.20
Ferulic acid	53.8 ± 0.4	0.12–17.0	*y* = 94 621*x* − 15 153	0.9993	0.004	0.01
Benzoic acid	272.5 ± 0.7	0.85–55.0	*y* = 9578.2*x* − 2819.6	0.9998	0.11	0.34
Eriodictiol	1.1 ± 0.002	0.33–21	*y* = 64 719*x* + 2777	0.9959	0.14	0.41
Cinnamic acid	25.6 ± 0.98	0.02–7.0	*y* = 24 5305*x* + 7475.1	0.9998	0.15	0.45
Luteolin	5.4 ± 0.3	0.13–17	*y* = 67 131*x* − 11 529	0.9992	0.020	0.06
Kaempferol	4.3 ± 0.2	0.05–15.0	*y* = 70 120*x* + 6037.1	0.9996	0.02	0.06
Apigenin	5.5 ± 0.1	0.17–11.0	*y* = 95 601*x* − 6571.1	0.9997	0.03	0.10

## Discussion

The HET-MN method is a rapid, simple, and cost-effective approach for determining genotoxic effects and it has some advantages. During the incubation period when this method is applied, the chicken embryo has not yet initiated brain activity. However, it possesses a high degree of metabolic activity. Due to this characteristic, the HET-MN system is considered superior to non-animal testing methods. The perception that HET-MN is closer to *in vitro* tests and is not an animal experiment is considered reasonable from both ethical and animal-rights perspectives. One of the major advantages of this test is that it allows the substance being tested for genotoxicity to pass through systemic stages such as Absorption, Distribution, Metabolism, and Excretion (ADME).^12–14^ During the incubation period when HET-MN is applied, nerves do not contact the chorioallantoic membrane, and pain receptors have not yet developed. Additionally, the living environment of the embryo within the egg is close to physiological conditions. The composition of the circulatory system in chicken embryos resembles that of erythrocyte cell composition in the bone marrow of adult mammals. Moreover, the ratio of polychromatic erythrocytes in the peripheral blood is 5% in mice, whereas it is 25–50% in a chicken embryo on the 11th day of incubation. The spleen, which is present in mammals, has not yet developed in a chicken embryo on the 11th day of incubation, and it eliminates abnormal erythrocytes. In embryos on the 11th day of incubation, due to the undeveloped spleen, erythrocytes containing MN accumulate in the circulation. This allows for more accurate results with the HET-MN method. This method also facilitates the evaluation of nuclear abnormalities. Data can also be easily analyzed by automating the process with a computerized image analysis system. While it cannot, of course, always be an alternative to mammalian test systems alone, it can reduce the number of animals and trials involving mammals.^7,9–11^ Studies conducted using the HET-MN method have been steadily increasing, and this test is gaining more importance with each passing day.^8,9,11–13,16,19–25^ Greywe *et al.*^8^ tested a wide range of genotoxic and non-genotoxic substances using HET-MN at two locations: Osnabrueck University and Henkel AG & Co., and obtained consistent results from HET-MN at both sites. These researchers also reported that their findings were consistent with those obtained using both *in vivo* and *in vitro* testing systems and concluded that the HET-MN method is a promising complement to existing testing methods. Hothorn *et al.*^20^ proposed a statistical model using a modified Williams-type procedure with Freeman–Tukey square-root transformed data for HET-MN testing.

It has been reported that substances to be tested using HET-MN can be injected into the air chamber of eggs on day 8 of incubation, and blood samples can be collected from embryos on day 11 of incubation. In many studies, at least three different doses of the test substance, each at 50 µl, 100 µl, 150 µl, 200 µl, 300 µl or 1000 µl for water-soluble compounds, were preferred. It was also suggested that at least 6 eggs should be used per dose, and at least 1000 cells should be evaluated per egg.^8–11,13,14,20^ Therefore, in our study, we used 100 and 200 µl test substance volumes and evaluated 5000 cells.

CP is an alkylating agent that has been widely used as a genotoxic substance in both *in vivo* and *in vitro* genotoxicity studies.^7,10,11,25–31^ We used 50 µg per egg of CP as the genotoxic substance, as recommended for HET-MN by others.^8–11^ Wolf *et al.*^10^ reported that the indicator of genotoxicity is the frequency of micronucleated E II including normo- and polychromatic cells (MNE II) in HET-MN. In their study, Wolf *et al.*^10^ reported the MNE II frequency in the positive control group as 13.2 ± 4.9‰, 7.3 ± 4.0‰, and 9.3 ± 1.4‰, respectively. In our study, this frequency was higher (2.29 ± 0.12%). This difference may be due to variations in the chicken breeds or hybrids and egg weights. While other HET-MN studies ^9–11^ used White Leghorn strain Lohmann selected (LSL) eggs weighing 65 ± 5 g, we used ATABEY hybrid breeder eggs weighing 60.49 ± 2.08 g.

AA is an antioxidant.^32,33^ The antimutagenic property of AA may be related to its ability to block the covalent binding of alkylating agents to cellular DNA.^34^ AA is commonly used as an antigenotoxic agent *in vivo*, *in vitro* and *in ovo* studies.^16,33–41^ Therefore, we used 50 µg per egg AA to assess its antigenotoxic effects using the HET-MN method. AA significantly reduced CP-induced MN formation in the CA group. We found that 50 µg per egg AA was effective in exhibiting antigenotoxic properties.

Various studies in the literature have investigated the genotoxic effects of propolis. Özkul *et al.*^42^ examined the anticancerogenic effects of propolis *in vitro* in human lymphocytes. The researchers concluded that propolis, tested at different concentrations (0.01 ml, 0.05 ml, 0.1 ml, 0.2 ml, 0.5 ml, 0.7 ml, and 1.0 ml), did not induce a carcinogenic effect in peripheral human lymphocytes. However, increased MN frequencies suggested that propolis might exhibit carcinogenic effects at higher concentrations. Tavares *et al.*^43^ studied the genotoxicity of Brazilian green propolis in Chinese hamster ovary cells. They evaluated parameters such as the Mitotic Index (MI) and the frequency of chromosomal aberrations. The researchers observed a slight but significant increase in the frequency of chromosomal aberrations at the highest propolis dose. However, the lowest dose of propolis significantly reduced chromosomal damage. Özkul *et al.*^44^ investigated the genotoxic effects of Bursa (Turkiye) propolis on human lymphocytes (*in vitro*) using the Sister Chromatid Exchange assay (SCE). They incubated blood samples from 10 healthy volunteers who did not use alcohol or smoke and exposed them to increasing concentrations of propolis (5 mg ml^−1^, 25 mg ml^−1^, 50 mg ml^−1^, and 250 mg ml^−1^). According to the results, increased SCE frequencies confirmed that Bursa propolis could exhibit genotoxic effects at high doses. Pereira *et al.*^45^ tested the genotoxicity of Brazilian green propolis in peripheral blood cells of mice using the Single Cell Gel Electrophoresis Assay (Comet assay) and MN test. Both MN and Comet tests showed that Brazilian green propolis induced a genotoxic effect in blood cells. The researchers found that acute consumption of Brazilian green propolis caused a mutagenic effect in the peripheral blood cells of mice. Senedese *et al.*^46^ aimed to evaluate the potential mutagenic effects of topical formulations supplemented with green propolis extract (1.2%, 2.4%, and 3.6%), used in the treatment of burns, in Chinese Hamster Ovary cells (*in vitro*) and Wistar rats (*in vivo*) through chromosomal abnormality analysis and the MN test. They found that the topical formulations containing green propolis in different concentrations did not exhibit mutagenic effects in either test system, although the 3.6% propolis gel was cytotoxic in the *in vitro* test. Montoro *et al.*^47^ evaluated the genotoxic effects of ethanol-propolis extract at increasing concentrations (0–2000 µg ml^−1^) *in vitro* on human lymphocytes. The researchers observed that propolis significantly reduced MI and Proliferation Index (PI) at higher concentrations. The increased SCE frequencies indicated that propolis might have genotoxic effects at high concentrations. Bayram *et al.*^48^ aimed to determine the genotoxic and antigenotoxic effects of propolis (0.2 mg per petri, 0.4 mg per petri, 0.6 mg per petri, 0.8 mg per petri, and 1.0 mg per petri) using Bacterial Reverse Mutation Test (Ames test). According to the viability test results, propolis was highly toxic at concentrations above 1.0 mg per petri, whereas it showed no toxicity at lower concentrations. Genotoxicity test results indicated that propolis at concentrations up to 1.0 mg per petri did not exhibit any genotoxic effects in *Salmonella typhimurium* strains TA1535 and TA1537, or in *E. coli* WP2uvrA. Antigenotoxicity test results showed that propolis exhibited significant antigenotoxic effects against mutagenesis induced by NaN_3_, 9-AA and MNNG on the same strains. Cruz *et al.*^49^ aimed to determine the antioxidant effect of the ethanol extract of Portuguese propolis and its dose-dependent genotoxic and antigenotoxic effects using the Comet assay. The results obtained from the Comet assay indicated that propolis was antigenotoxic at lower concentrations and genotoxic at higher concentrations. They suggested that this dual effect was associated with the presence of antioxidant compounds such as kaempferol, pinobanksin, and pinocembrin, as well as compounds like caffeic acid phenethyl ester (CAPE) and chrysin, which inhibit DNA synthesis and cell proliferation. In our study, the HET-MN results showed that water-based organic Turkish propolis did not exhibit genotoxic effects at any of the three doses tested (500 µg per egg, 250 µg per egg and 50 µg per egg). To determine the antigenotoxic effect, high-dose propolis (500 µg per egg) showed a synergistic effect when applied with CP, enhancing the effect of CP. The medium-dose propolis (250 µg per egg) reduced the effect of CP, while low-dose propolis (50 µg per egg) significantly reduced the effect of CP and exhibited antigenotoxic activity.

The Janus pathway is a response mechanism in which some compounds show dual effects in cells, one positive and one negative. Some compounds may exhibit genotoxic effects *via* the Janus pathway, whereas under altered conditions they may exhibit antigenotoxic effects. Several studies have shown that many chemicals may demonstrate genotoxic effects in one tissue type while exhibiting antigenotoxic effects in another. The dual effects of compounds in the Janus pathway can vary across cell types, test-substance doses, and exposure durations. Many flavonoids in propolis exhibit dual effects on the Janus pathway.^3,50^ Bozkuş *et al.*^51^ produced water-based organic Turkish propolis from raw propolis collected from four different regions of Turkiye. When they analyzed the content of the product using HPLC, they detected chlorogenic acid at a concentration of 10.20 mg µl^−1^, caffeic acid at 204.00 mg µl^−1^, 3,4,5-tri-*O*-caffeoylquinic acid at 7.75 mg µl^−1^, and *trans*-cinnamic acid at 28.90 mg µl^−1^. In this study, benzoic acid and caffeic acid were detected at high concentrations in the chemical composition of water-based organic Turkish propolis, as well as some flavonoids in lower concentrations. Therefore, the fact that water-based organic Turkish propolis increased the genotoxicity of CP at high doses while reducing it at low doses, indicating antigenotoxic effects, may be related to the responses observed with benzoic acid (272.5 µg ml^−1^) and caffeic acid (265.4 µg ml^−1^) in the Janus pathway. Various studies have proven that caffeic acid, one of these compounds, exhibits genotoxic effects at high doses.^52^ Benzoic acid and caffeic acid are antioxidants from the phenolic acid group, and as reported in many studies, the activities of natural antioxidants are closely related to their ability to reduce DNA damage, mutagenesis, and carcinogenesis. However, some antioxidants have also been reported to cause DNA damage, depending on their concentration.^53^

Fikri *et al.*^54^ aimed to examine the effects of propolis administration during pregnancy on fetal development. To this end, they administered aqueous and ethanolic propolis extracts at different doses to pregnant rats. According to the researchers' findings, the ethanolic and aqueous propolis extracts did not hinder fetal development at low doses, but at high doses they significantly reduced fetal weight. Similarly, in our study, the water-based organic Turkish propolis reduced embryo weight. Additionally, the researchers stained the fetuses' bones with Alizarin Red S to examine skeletal development. Their results showed that the high dose of the ethanolic propolis extract significantly reduced bone thickness, whereas the high dose of the aqueous extract caused a moderate reduction. In our study, the water-based organic Turkish propolis did not exhibit any macroscopic adverse effects on the skeletal development of chicken embryos. Dawson's technique is an anatomical technique which diaphanizes soft tissues and stains embryo bones.^55^ Several modifications have been made since the first description of the technique and it is considered as a very useful technique for studying the embryological development of the skeletal system. Essentially, the method consists of macerating the fetus with alkali, followed by staining it with a dye, which has a strong affinity for calcium salts, such as Alizarin Red-S (a sulfonate form of a dye derived from anthraquinone). The resulting specimens allow the visualization of the stained red bone beneath the transparent soft tissues.^15^ In this study, Alizarin Red-S staining revealed that the primary ossification centers, particularly in the diaphysis of the upper and lower extremity bones (humerus, radius-ulna, carpometacarpus/femur, tibiotarsus, tarsometatarsus) appeared red. No macroscopic differences were observed in these areas especially when comparing the experimental and control groups.

Liu *et al.*^56^ examined the effects of caffeic acid on reproductive and developmental toxicity in mice. In their study, the researchers administered 0.15, 5, and 150 mg per kg per day doses of caffeic acid *via* gavage to pregnant mice. They observed that administering 5 and 150 mg per kg per day of caffeic acid affected embryo implantation when administered before the sixth day of pregnancy. They also observed a reduction in fetal weight at the 150 mg per kg per day dose. No maternal toxicity, fetal teratogenesis, or postnatal effects were observed. Similarly, in our study, the water-based organic Turkish propolis reduced embryo weight but did not show teratogenic or embryotoxic effects. This could be due to the high concentration of caffeic acid in the water-based organic Turkish propolis, as reported by Bozkuş *et al.*^51^

## Conclusion

According to the results of the HET-MN test, water-based organic Turkish propolis did not exhibit any genotoxic effects at any of tested doses. To evaluate its antigenotoxic potential, propolis at the highest dose was co-administered with CP, resulting in a synergistic interaction that enhanced CP's genotoxic effect. CP is an alkylating antineoplastic drug used to treat cancer patients during chemotherapy; however, it also has toxic effects such as carcinogenicity and mutagenicity. Therefore, caution should be exercised when using propolis in patients undergoing chemotherapy, particularly for purposes such as enhancing the body's resistance and supporting bodily functions. Notably, the lowest dose propolis significantly decreased the genotoxic effect of CP, demonstrating a statistically significant antigenotoxic effect. Some compounds can both induce and prevent damage. Therefore, if propolis is to be used to prevent diseases such as cancer, it is crucial to first understand the conditions under which it supports health and prevents genomic damage. To avoid unwanted harmful effects of propolis, dietary supplements and medical products containing propolis should be carefully formulated and biologically studied. Due to the high concentration of caffeic acid in water-based organic Turkish propolis as reported above and its ability to reduce the weight of live embryos, care should be taken when using propolis in pregnant women. It should be used under medical supervision or even avoided. Given the effects of propolis during the embryonic period, low doses are relatively safe, whereas high doses require caution. In this study, three different doses of water-based organic Turkish propolis were tested using HET-MN. To better understand its effects, additional tests with different doses are required.

## Ethical statement

This study was approved by Selçuk University, Faculty of Veterinary Medicine Experimental Animals Production and Research Center Ethics Committee (Approval No. 2022/08).

## Author contributions

Conceptualization, SA, HÖ.; methodology, SA, HÖ.; software, SA, HÖ, GZ.; validation, HÖ, GZ.; formal analysis, SA, HÖ.; investigation, SA, HÖ.; resources, HÖ.; data curation, SA, HÖ, GZ.; writing—original draft preparation, SA, HÖ, GZ.; writing—review and editing, HÖ, GZ.; visualization, HÖ.; supervision, HÖ.; project administration, HÖ.; funding acquisition, HÖ. All authors have read and agreed to the published version of the manuscript.

## Conflicts of interest

No potential conflict of interest was reported by the authors.

## Data Availability

Data will be requested from authors.
